# Exploring the Effect of Cooperation in Reducing Implicit Racial Bias and Its Relationship With Dispositional Empathy and Political Attitudes

**DOI:** 10.3389/fpsyg.2020.510787

**Published:** 2020-10-28

**Authors:** Ivan Patané, Anne Lelgouarch, Domna Banakou, Gregoire Verdelet, Clement Desoche, Eric Koun, Romeo Salemme, Mel Slater, Alessandro Farnè

**Affiliations:** ^1^INSERM U1028, CNRS U5292, Integrative Multisensory Perception Action & Cognition Team (ImpAct), Lyon Neuroscience Research Center, Lyon, France; ^2^Claude Bernard University of Lyon 1, Lyon, France; ^3^Hospices Civils de Lyon, Mouvement et Handicap, Neuro-Immersion, Lyon, France; ^4^Event Lab, Department of Clinical Psychology and Psychobiology, University of Barcelona, Barcelona, Spain; ^5^Institute of Neurosciences, University of Barcelona, Barcelona, Spain; ^6^Center for Mind/Brain Sciences, University of Trento, Trento, Italy

**Keywords:** cooperation, virtual reality, embodiment, implicit racial bias, empathy, implicit association test, political attitudes, inclusion of the other in the self

## Abstract

Previous research using immersive virtual reality (VR) has shown that after a short period of embodiment by White people in a Black virtual body, their implicit racial bias against Black people diminishes. Here we tested the effects of some socio-cognitive variables that could contribute to enhancing or reducing the implicit racial bias. The first aim of the study was to assess the beneficial effects of cooperation within a VR scenario, the second aim was to provide preliminary testing of the hypothesis that empathy and political attitudes could contribute to implicit bias about race, while the third aim was to explore the relationship between political attitudes and empathy. We had (Caucasian) participants embodied in a Black virtual body and engaged either in a cooperative (Coop group) or in a non-cooperative (Neutral group) activity with a confederate experimenter embodying another Black avatar. Before and after VR, we measured participants’ implicit racial bias by means of Implicit Association Test (IAT) and their perceived closeness toward the confederate experimenter. Before VR we also assessed participants’ political attitudes and empathy traits. Results revealed that, as compared to the Neutral group, the Coop group showed lower IAT scores after the social interaction. Interestingly, in the Neutral but not the Coop group the perceived closeness toward the confederate experimenter was associated with the initial racial bias: the more the participants reduced their distance, the more they reduced their IAT score. Moreover, reported traits of empathy and political attitudes significantly explained the variance observed in the initial implicit bias, with perspective-taking, empathic concern, and personal distress being significant predictors of the IAT scores. Finally, there was a relationship between political attitudes and empathy: the more participants considered themselves as left-wing voters, the higher their perspective-taking and empathic concern scores. We discuss these findings within the neuroscientific and social cognition field and encourage scholars from different domains to further explore whether and under which conditions a given manipulation for reducing racial bias could be efficiently transposed in VR.

## Introduction

Several studies have shown how virtual reality (VR) can be used as a powerful tool to alter one’s brain-body representation. Inspired by the rubber hand illusion (Botvinick and Cohen, 1998), such paradigms have demonstrated that under the necessary multisensory conditions, a person’s real body or body part can be substituted with a virtual life-sized one (Sanchez-Vives et al., 2010; Slater et al., 2010; Normand et al., 2011; Kilteni et al., 2012), resulting in the perceptual illusion of *body ownership* (Ehrsson, 2007; Lenggenhager et al., 2007; Blanke, 2012; Ehrsson, 2012; Blanke et al., 2015). Beyond embodying the physical avatar, it is now well-established that attributes of the surrogate body can have an impact on people’s perception, attitudes, and even higher cognitive capabilities (Maister et al., 2015; Osimo et al., 2015; Banakou et al., 2018; Beckerle et al., 2019; D’Angelo et al., 2019; Ventre-Dominey et al., 2019). Yee and Bailenson (2007) referred to this as the Proteus Effect, suggesting that behaviors can be influenced by assumptions about how one would believe others would expect them to behave. They reported, for example, that when the virtual face of participants appears to be more attractive than their real one, it alters their proxemics behavior, or that when participants are embodied in a taller virtual body they will become more aggressive in negotiation than when in a shorter virtual body. Another study showed that embodiment of adults in a child’s virtual body results in over-estimation of object sizes and implicit self-identification as being child-like (Banakou et al., 2013); these findings having been replicated in Tajadura-Jiménez et al. (2017). More recently, Banakou et al. (2018) showed that embodying virtual Albert Einstein results in better performance on an executive functions task, as compared to embodying a normal-looking body, and also reduces implicit bias against the elderly, a result first observed in Yee and Bailenson (2006). Similarly, Osimo et al. (2015) and Slater et al. (2019) showed that when participants find themselves in the virtual body of Dr. Sigmund Freud, it helped them find a more satisfactory solution to a personal problem and positively influenced their mood compared to when they are embodied in a look-alike virtual body.

Such embodiment techniques have been used in the attempt to address the malleability of the implicit racial discrimination, in various contexts. In the rubber hand illusion, for example, ownership over a dark-skinned rubber hand has been found to reduce implicit bias in Caucasian participants (Maister et al., 2013; Farmer et al., 2014), and that ownership itself arises regardless of implicit attitudes toward that race. In the field of virtual embodiment, Groom et al. (2009) first addressed racial discrimination by placing participants in a Black virtual body, which they experienced from a first-person perspective (1PP) with synchronous head movements. Participants were placed in a job interview setting, and the results showed an increase in implicit racial bias of White participants against Black people. Later findings, however, found the opposite effect. Peck et al. (2013) used virtual embodiment over a full virtual body with synchronous visuomotor correlations and showed a reduction of implicit bias. Importantly, for the development of potential societal applications, the same effect was reported to last (at least) 1 week after participants’ exposure to a similar VR interaction in Banakou et al. (2016). Salmanowitz (2018) examined the influence of embodying a different race body on implicit racial biases within legal scenarios. After embodying a Black virtual body, participants had lower implicit racial bias scores as compared to those who were immersed in a sham embodiment condition, whereby only the handheld controllers, instead of their arms, were visible as they moved in the virtual space. Interestingly, they also evaluated an ambiguous legal case more cautiously, rating, for example, vague evidence as less indicative of guilt. The above examples have mainly relied on Implicit Association Tests (IAT) (Greenwald et al., 1998) to measure implicit bias, where essentially, participants are faster categorizing Black faces with negative attitudes and White faces with positive attributes than the opposite combinations. Instead, Hasler et al. (2017) studied the behavioral effects of embodying a White vs. a Black virtual body. It was found that this embodiment technique enhances mimicry toward the in-group when it comes to dyadic interactions, regardless of the type of the embodied virtual body. In other words, when embodied in Black bodies, White participants treat Black as the novel in-group, which then reverses bias. Some of these findings have been reviewed in Maister et al. (2015) and have been successfully simulated by a neutral network model proposed in Bedder et al. (2019).

In this study we aimed to expand on previous results by addressing the question of whether engaging in cooperative activity with an avatar of the same embodied ethnicity could contribute to reducing the implicit racial bias in dyadic interactions. It is evident from the studies of Groom et al. (2009) and Hasler et al. (2017) that the type of social interaction that participants are exposed to can have an influence on how implicit discrimination is modified. It has been suggested that the results obtained in Groom et al. (2009) were most likely related to the fact that the virtual scenario depicted a negative situation linked to racial discrimination and included multiple features invoking negative stereotypes (Blommaert et al., 2012). In fact, Hasler et al. (2017) reported that although IAT scores did not change as a result of the embodiment, i.e., mean changes were the same whether embodied in the White or Black body, for those embodied as Black the extent to which they liked their virtual partner was associated with a reduction in implicit bias. In other words, when embodied in a Black body, Caucasian participants who happened to have liked their virtual partner showed a greater reduction in implicit bias than those who did not. Not only the extent to which participants liked the other, but also the extent to which they perceived psychological closeness toward the other had a significant impact on implicit bias (where perceived closeness was assessed only after embodying a White or Black body). The authors concluded that participants could have experienced the social interaction as negative or positive depending on their own individual preferences. This could be a potential limitation of this technique: if participants experience the interaction as negative or do not perceive the other as close to themselves, the embodiment of a Black avatar could not be sufficient to reduce their initial racial bias. One could speculate that, as participants do not simply “like” the partner, the reduction of their implicit bias following the embodiment of a Black avatar could not be generalized to other members of the target outgroup, i.e., the category “Black,” as tested in IAT.

Capitalizing on this, here we aimed at testing whether (1) the social perception of the other person, in terms of psychological closeness, is critical in reducing implicit racial bias following the embodiment of a Black avatar and (2) whether a cooperative interaction with the virtual partner could induce a change of the initial racial bias, taking into account the potential effect of psychological closeness. To do so, we measured psychological closeness both before and after VR exposure, taking this difference as an index of social perception of the other, and examined the effect of a cooperative vs. neutral (non-cooperative) interaction on implicit racial bias. During the VR exposure, Caucasian participants embodied a Black body avatar facing another Black avatar and engaged in solving a 3D puzzle task either by themselves (neutral presence) or by cooperating with the Black avatar partner (cooperative presence). Here the neutral presence of the partner is similar to the interaction tested in Hasler et al. (2017), where participants and the virtual partner did not have to cooperate to achieve their goal. We could therefore predict several alternative scenarios. First, participants could reduce IAT scores more when cooperating than when having to do the task on their own, with such an effect being independent of any change in the perceived closeness toward the other person. Second, the decrease in IAT could be explained only by the perceived closeness, independently of the type of the interaction (i.e., the more one is close to the other, the more one reduces IAT, regardless of the interaction). Lastly, based on Hasler et al. (2017), we could predict a more articulated yet interesting scenario: within the context of a cooperative interaction, participants could show a reduction from the initial IAT after cooperating with such an effect being, at least partially, independent of any change in perceived closeness. By contrast, within the context of a neutral interaction, the changes in IAT could be associated with perceived psychological closeness, similarly to effect of likability in Hasler et al. (2017).

In addition to assessing the role of cooperation and psychological closeness and their potential interaction on the implicit racial bias change, this study also aimed at exploring the putative role of other factors in determining such a bias. If personality psychology studies suggest that prejudice and racial attitudes are functions of people’s personality characteristics such as social dominance orientation (Pratto et al., 1994), while social cognition and neuroscience stress the role of empathy (Avenanti et al., 2010), only very few studies have explored a social variable that has a massive impact on our everyday life: political attitudes. In particular, here we tested whether dispositional empathy and political attitudes could be associated with initial racial bias, before any embodiment manipulation. Finally, we assessed the relationships between social dominance orientation, dispositional empathy, and political attitudes, as those variables could potentially represent important factors to be taken into account in future studies about embodiment and racial bias.

The study was conducted with 40 White male and female participants, who experienced themselves embodied in a Black virtual body seen from 1PP and with full visuomotor synchrony, i.e., virtual body movements corresponded to participants’ actual body movements. This was a between-groups design with a single factor group with two levels (*Coop* vs. *Neutral*). In the Coop group participants and a virtual confederate had to complete the construction of the Colosseum 3D puzzle, while in the Neutral group participants had to complete the construction of the puzzle on their own, without any help from the Black confederate, who was still present in the virtual scenario and merely observed participants solving the task. Details about the form of cooperation and the experimental setup are given in Methods and can also be viewed in Supplementary Videos S1–S3.

## Materials and Methods

### Participants

Forty participants (13 men, 27 women, mean age = 25.8 years, *SD* = 5.46) with normal or corrected-to-normal vision and no history of neurological or psychiatric disorders took part in the study. They were randomly assigned to one of the two groups (Coop or Neutral). All participants were naive as to the experimental hypotheses and provided written informed consent. The study was approved by the Inserm ethics board (IRB00003888) and complied with the ethical standards outlined in the Declaration of Helsinki (World Medical Association, 2013).

### Procedures

**FIGURE 1:**
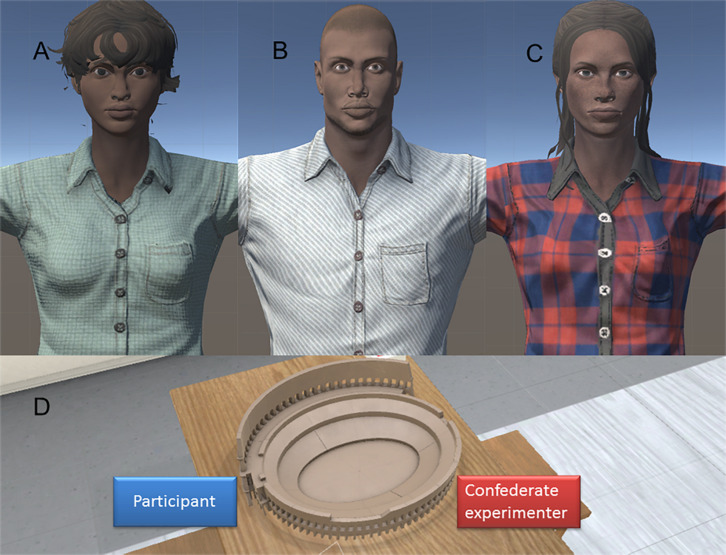
The virtual bodies for **(A)** female participant, **(B)** male participant, and **(C)** confederate experimenter. **(D)** Model of the Colosseum participants had to build in the presence of the confederate experimenter.

Before the VR exposure, participants completed a series of questionnaires assessing political attitudes, empathy, and social dominance orientation (see below). Then they completed a racial bias Implicit Association Test (IAT, hereafter preIAT) and the Inclusion of the Other in the Self, IOS, scale (preIOS, see below), their order being counterbalanced between participants. After these pre-exposure measures, participants were asked to sit at a table and to wear the HMD and a motion capture suit. The position of the table corresponded to the center of the physical and virtual room. The virtual room was decorated with the same furniture as the real room with a virtual mirror on the left wall. During the initial familiarization phase of the experiment, participants were instructed to turn and move their head, trunk, and arms and look at themselves in the virtual mirror to explore the capabilities and real time motion of the virtual body (see Supplementary Video S1). The body of the participant was substituted by a same-sex Black virtual body, seen from 1PP. Therefore, participants could see their body both by looking directly toward their virtual body and also in the virtual mirror. The participant’s head and body movements were mapped to the virtual body. After this familiarization period (lasting about 3 min), participants were instructed that the confederate experimenter would enter the virtual room as a second virtual character sitting in front of them at the table. To note, participants were not informed that the confederate experimenter (a White female) would also be embodied in a Black avatar. In the cooperation group (Coop), participants and the confederate experimenter had to cooperate in order to solve a 3D puzzle of the Colosseum (see Figure 1). In particular, solving the puzzle task required the help of the other avatar because half of the bricks had to be positioned too distant to be placed alone. Participants were thus required to pass the bricks to the confederate experimenter so that they could be put in the right place, and vice versa (see Supplementary Video S2). In the other group (Neutral), the distant bricks were already positioned and participants had to complete the construction of the Colosseum on their own, while still facing the same avatar of the confederate experimenter (see Supplementary Video S3). The whole VR procedure lasted approximately 9 min. Then, the HMD was removed, and participants completed the IAT (postIAT) and the IOS (postIOS) again. Finally, we administered a series of post-exposure questionnaires about participants’ experience, including questions about the level of subjective ownership over the virtual body (see Banakou et al., 2016; Banakou et al., 2018), as well as the level of cooperation, difficulty, positivity, and pleasantness of the interaction with the confederate experimenter (see Patané et al., 2017).

### Virtual Environment and Avatar Animation

Participants were fitted with an Oculus Rift CV1 head-mounted display. This has two OLED displays (5.7 inches) with a 2160 × 1200 pixels resolution, so 1080 × 1200 per eye displayed at 90 HZ, and with a field of view of 110 degrees. Head tracking was performed by an Oculus sensor (tracker). The Headset and Tracker are connected to a workstation (PC unit) via USB cables. For a full product description^1^. Participants were also required to wear a Perception Neuron body motion capture suit tracking their upper body (as they sit at a table) from Noitom^2^ with 22 Neuron Sensors, inertial sensors to capture the motion data. Each sensor hosts an Inertial Measurement Unit (IMU) with a gyroscope, accelerometer, and magnetometer. A hub collects the motion data from the Neuron Sensors. It then sends the data to the computer via USB Perception Neuron motion capture system, which communicates with the Axis Neuron software application, which processes the motion data. For a full product description^3^. The experiment ran on two computers with Windows 10, an Intel(R) Core i7-8750 with a frequency 2,20 GHz and a graphics card Nvidia GeForce GTX 1060 connected on a local network. The virtual environment was created with 3D Studio Max 2018 and implemented on the Unity 3D platform (Unity 5.5.1). The environment consisted of a virtual version of the real experimental room, with a projector and its screen, a table with two chairs, and a virtual mirror reflecting the participant’s and the confederate experimenter’s virtual body. When participants entered the virtual environment, they were embodied in a life-sized virtual body that visually substituted their real body as seen from their own 1PP perspective. As they moved their real body, they would see their virtual body moving.

The three virtual bodies were created with Adobe Fuse CC: a female or male version of the Black avatar, according to the participants’ sex, and another female Black avatar for the confederate experimenter (the confederate was always the same female Black avatar). They had different clothes, faces, and hairstyle, but similar dark skin color and height (see Figure 1). They were animated using Axis Neuron software and their body movements were generated by recording participants’ and confederate experimenter’s actual body movements. Since they sat at a table, only their upper-body (from head to lower back) movements were tracked and mapped to the avatars.

## Measures

### Virtual Body Ownership Ratings

To examine subjective experience of ownership over the virtual body, a 7-point scale was used (also ranging from −3 to +3). More specifically, these questions were related to the strength of body ownership (MyBody: “I felt that the virtual body I saw when looking down at myself was my own body”; Mirror: “I felt that the virtual body I saw when looking at myself in the mirror was my own body”) and agency (Agency: “I felt that the movements of the virtual body were caused by my own movements”). Here we expect that the levels of body ownership and agency are the same between the two groups. The other two questions served as control (Features: “I felt that my virtual body resembled my own [real] body in terms of shape, skin tone, or other visual features”; TwoBodies: “I felt as if I had two bodies”; see Banakou et al., 2016).

### VR Experience Ratings

In order to assess how participants perceived the experience with the confederate, participants were asked to rate the session using a 7-point bipolar scale on the following dimensions: difficult vs. easy, unpleasant vs. pleasant, positive vs. negative, cooperative vs. not-cooperative. The scale varied from −3 to +3, with (0) indicating a neutral response on each question (see Patané et al., 2017). These questions served as an important manipulation check, as we predicted the scores to be comparable between the two groups, except for the cooperative dimension. In particular, we should expect a higher score in the cooperative group.

### Implicit Racial Bias: IAT

Implicit racial bias was measured by administering to participants the racial IAT (Implicit Association Test, Greenwald et al., 1998, 2003) right before and after VR exposure. The racial IAT measures racial bias by requiring people to quickly categorize faces (Black or White) and words (positive or negative) into groups. An implicit bias score is calculated from the difference in speed and accuracy between categorizing (White faces, positive words) and (Black faces, negative words) compared to (Black faces, positive words) and (White faces, negative words). Higher IAT score is interpreted as greater racial bias, i.e., longer reaction times and greater inaccuracies in categorizing black faces with positive words and white faces with negative words than black faces with negative and white faces with positive words. The IAT was completed on the same desktop computer screen both times. Since IAT scores tend to show slightly stronger associations corresponding to the pairings of the combined block that is completed first, to control for this effect, the order was counterbalanced between participants (Nosek et al., 2007; Banakou et al., 2016).

### Psychological Closeness: IOS

To measure psychological closeness toward the confederate experimenter, we administered the IOS scale. The Inclusion of the Other in the Self, IOS, scale (Aron et al., 1992; Paladino et al., 2010) consisted of seven Venn diagram-like pairs of circles that vary on the level of overlap between the self and the other. Participants had to select the pair of circles that best represents their relation with the confederate before and after the VR experience.

### Self-Reported Dispositional Empathy: IRI

To measure dispositional empathy, we used the Interpersonal Reactivity Index scale (IRI; Davis, 1983). IRI measures on a 5-step Likert-type scale various facets of empathy through four scales, of 7 items each:

1. The perspective-taking scale (PT) measures the reported tendency to adopt spontaneously the psychological point of view of others (e.g., “I sometimes try to understand my friends better by imagining how things look from their perspective”).
2. The fantasy scale (FS) measures the tendency to imaginatively transpose oneself into fictional situations (e.g., “When I am reading an interesting story or novel I imagine how I would feel if the events in the story were happening to me”).
3. The empathic concern scale (EC) assesses the respondents’ feelings of warmth, compassion, and concern for others (e.g., “I often have tender, concerned feelings for people less fortunate than me”).
4. The personal distress stress scale (PD) assesses self-oriented feelings of anxiety and discomfort resulting from tense interpersonal settings (e.g., “Being in a tense emotional situation scares me”).

### Self-Reported Political Orientation

We assessed political attitudes by having participants rate themselves from strong extreme left (−3) through to extreme right (+3) on a Likert scale.

### Social Dominance Orientation

The social dominance orientation (SDO) scale measures one’s degree of preference for inequality among social groups and the extent to which one desires that one’s in-group dominate and be superior to out-groups. The French translation of the SDO scale (Duarte et al., 2004), originally constructed by Pratto et al. (1994), consists of 16 items. Some item examples:

– Some groups of people are just inferior to others (agreeing suggests high social dominance).
– We would have fewer problems if we treated all groups equally (agreeing suggests low social dominance).

## Results

### Virtual Body Ownership Ratings and VR Experience Ratings

The box plots of the questionnaire about subjective feelings of ownership over the virtual body show median scores above 0 in both groups for the first and second questions directly assessing ownership. Additionally, the two groups reported low scores in the two control questions. The box plots also suggest that participants had a comparable experience of ownership toward the Black virtual body across the Neutral and Cooperative groups. This is in line with all previous findings.

**FIGURE 2:**
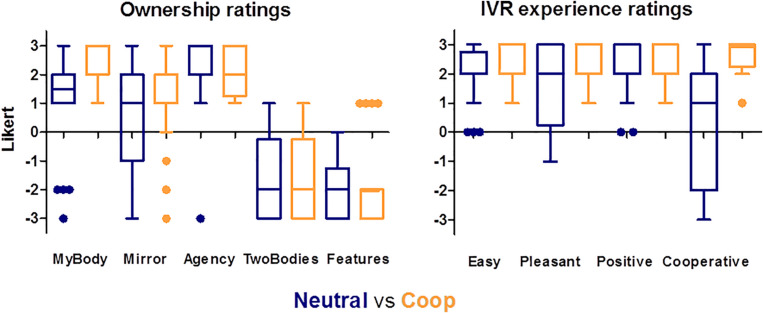
Box plots of body ownership ratings (left panel) and of perception of the experience in VR (right) by group (Neutral in blue, Coop in orange) showing median and the interquartile ranges with Tukey whiskers. Individual points are outliers.

Finally, with regards to the questionnaires exploring the subjective perception of the VR experience, the participants in the two groups reported very high scores (medians between 2 and 3) with the only exception of the cooperation dimension for the Neutral group. The box plots also suggest that the participants in the Coop group experienced the interaction in VR as being more cooperative than the Neutral group (see Figure 2, left right), supporting the effectiveness of our manipulations.

### Effects of Cooperation and Psychological Closeness on Implicit Racial Bias

**FIGURE 3:**
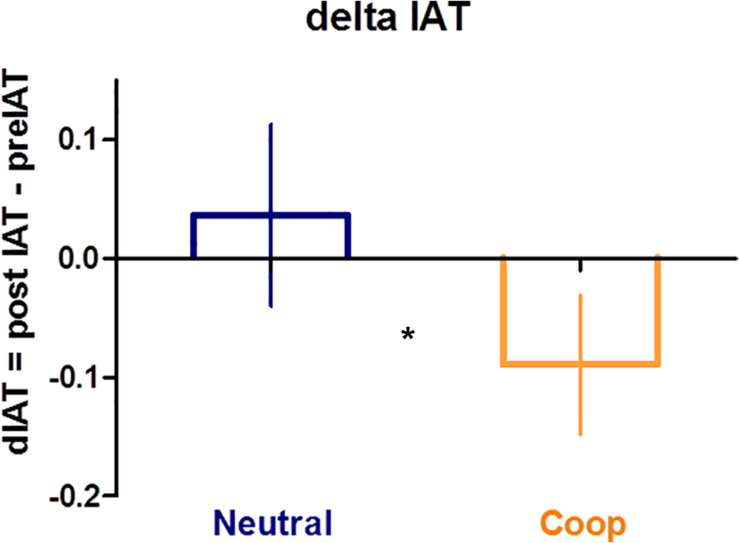
Bar chart displays means and standard errors of dIAT by group (Neutral in blue, Cooperative in orange). Asterisk denotes a significant difference between Neutral and Coop.

**FIGURE 4:**
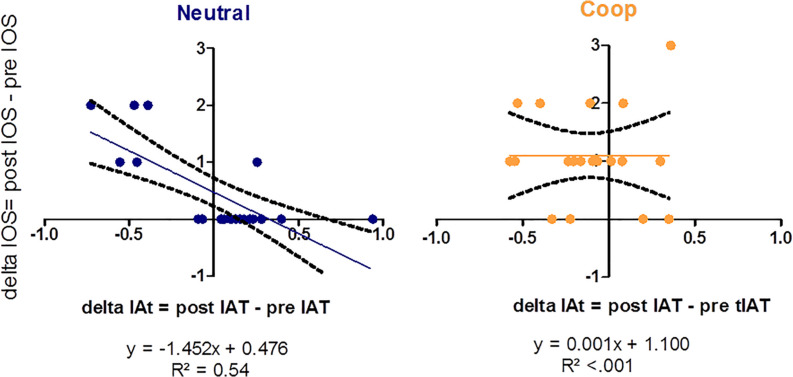
Scatter plots (with best-fitting regression lines + 95% CI) showing delta IAT, separately for the Neutral and Coop group, as a function of delta IOS.

Since preliminary analysis revealed that the VR exposure modulated the perceived closeness toward the other person (see Supplementary Analyses S1), we tested whether the change in IOS could also impact on the change in IAT scores observed in the two groups. To assess the potential effect of psychological closeness on implicit racial bias, we fitted a linear model (ANCOVA) to submit data to inferential analysis. A Repeated Measures ANCOVA was conducted on the dIAT (calculated prior vs. following the VR exposure), taking the group (Coop vs. Neutral) as between-subject factor. To test for the influence of psychological closeness, we entered dIOS scores as a covariate. Finally, we also included in the model the two-way interaction as well as the two main effects. Results revealed a significant main effect of the dIOS, *F*(1,36) = 9.97, *p* = 0.003, $\eta_{\text{p}}^{2}$ = 0.22, and a significant main effect of group, *F*(1,36) = 5.15, *p* = 0.029, $\eta_{\text{p}}^{2}$ = 0.13 with lower dIAT in Coop (−0.11 ± 0.06) as compared to Neutral (0.02 ± 0.08, see Figure 3). Moreover, Group and dIOS significantly interacted *F*(1,36) = 9.99, *p* = 0.003, $\eta_{\text{p}}^{2}$ = 0.003. Figure 4 shows that in the situation of neutral experience with the confederate experimenter there was a negative association between dIAT and dIOS. By contrast, in the case of the cooperative interaction there was no association between dIAT and dIOS.

To sum up, the analyses showed that, as compared to the Neutral, the Cooperative group reported a significant reduction in the initial IAT score and such a reduction was not modulated by the difference in the IOS score. On the contrary, in the Neutral group the difference in the IAT score was negatively associated with the difference in the IOS score: the higher the closeness after VR exposure, the lower the IAT after VR exposure.

### Initial Implicit Racial Bias

To investigate the factors that could contribute to the implicit racial bias, we computed a multiple regression model on the preIAT scores, i.e., before any experimental manipulation, collapsing the two groups. Specifically, we focused on the four scales of IRI and political party preference. The results of the regression indicated that the model explained 38.4% of the variance and that the model was a significant predictor of preIAT, *F*(1,39) = 4.24, *p* = 0.004. Although political preference (*B* = −0.006, *p* = 0.92) and FS scale (*B* = 0.027, *p* = −207) did not contribute significantly to the model, PD (*B* = 0.053, *p* = 0.002), EC (*B* = −0.057, *p* = 0.026) and PT scale (*B* = −0.035, *p* = 0.044) significantly predicted implicit racial bias. To note here, PD was positively associated, while EC and PT were negatively associated with preIAT.

### Explorative Correlations: Left-Wing Voters Are More Emphatic

Finally, as a further aim of this study was to examine any mutual relationships between social dominance orientation, empathy, and political orientation, we carried out a series of correlations, corrected for multiple comparisons between SDO, IRI scales, and political party preference. SDO was positively correlated with political party preference (*r* = 0.49, corrected *p* = 0.014) and negatively with FS scale (*r* = −0.49 corrected *p* = 0.014) and EC scale (*r* = −0.51, corrected *p* = 0.007). Interestingly, there was a negative relationship between political party preference and PT scale (*r* = −0.45, *p* = 0.036). Finally, political party preference negatively correlated with EC (*r* = −0.56, *p* = 0.001).

## Discussion

This study aimed at testing the potential effect of a cooperative interaction in reducing implicit racial bias after embodying an avatar of a different ethnicity, as well as exploring the contribution of other social factors, namely dispositional empathy and political attitudes, in determining such a bias and their mutual relationship. First, the VR ratings support the effectiveness of our embodiment procedure. Indeed, the three manipulation check questions directly assessing ownership and agency had high scores in both groups, thus suggesting that (all Caucasian) participants had strong body ownership illusion over the Black avatar. This is in line with previous results on full-body ownership in VR in indicating that experiencing the body from 1PP, as well as visuomotor synchrony of the virtual movements with the real ones, are critical factors for body ownership (Sanchez-Vives et al., 2010; Slater et al., 2010; Normand et al., 2011; Kilteni et al., 2012; Banakou et al., 2013). Second, the ratings assessing the qualitative aspects of the social interaction indicated that the cooperative presence of the other was also effective. The interaction was indeed perceived as more cooperative than in the neutral group, while the social perception of the interaction in terms of difficulty, positivity and pleasantness, appeared comparable between the two groups.

Here, we focus on the first aim of this study. For testing our main hypothesis about the impact of cooperation, we compared the IAT scores before and after a cooperative (Coop group) or neutral (Neutral group) presence of a confederate experimenter. Additionally, we measured psychological closeness, through the IOS scale, toward the confederate experimenter, as we hypothesized this social dimension, though independent of the VR embodiment procedure, could impact the IAT. The results revealed that the initial implicit bias decreased more for those who cooperatively interacted, as compared to those who were engaged in a neutral situation with the confederate experimenter. It was previously argued in Banakou et al. (2016) that negative derived associations with the concept “Black,” due to our social environment (and especially the media), are reflected in the IAT despite people’s explicit attitudes. Based on how the IAT operates, when a White person is embodied in a Black body, this novel embodiment information disrupts and updates the previously formed negative associations, as there is a new piece of evidence: “I” have Black skin. This new piece of evidence is an important one as it is associated with one’s Self, which carries a whole new set of associations that are more likely to be positive than negative. This argument was analogous to that of Maister et al. (2015), that similarity between appearance of the self (during body ownership) and the out-group results in the disruption of associations between the out-group and negative valence items, and substituted by positive associations with the self. These findings have been formalized in a neural network model and theory developed in Bedder et al. (2019). Complementary findings come from a recent fMRI study investigating whether taking a first-person embodied perspective during the experience of domestic violence enhances the identification with the victim (de Borst et al., 2020). The authors found that 1PP exposure in VR, which is similar to our embodiment technique, increased body ownership and identification with the virtual victim. This is accompanied by synchronization in the fronto-parietal network associated with the bodily Self to predict actions toward the body and in the amygdala to signal the proximity of the stimulus in the nearby space. Here we speculate that when the embodiment in 1PP is associated with a positive experience (i.e., like in a cooperative interaction), the similarity between the new-acquired image of the bodily Self (through body ownership) and the out-group’s member could result in a downregulation of the amygdala activity. This could also explain the decreased IAT scores we observed after cooperation. On the contrary, when embodiment is associated with a negative experience (like in de Borst et al., 2020), the overlap between the bodily Self and the outgroup results in an increased activity of the amygdala. While this neuro-anatomical model needs to be thoroughly tested, here we argue that several variables can promote the overlap between the Self and outgroup induced by the embodiment of a Black body. Indeed, the type of activity (cooperative or not) is only one of the many variables that can facilitate a new set of positive associations following the virtual embodiment manipulation. Specifically, future work should take into account many other situational and dispositional moderators, such as trustworthiness, emotional intelligence and contagion, cognitive closeness, positive and rewarding experience with the other, tendency to fairly behave, observation of the others’ struggle and pain, repeated contact to the out-group members, or egalitarian (or anti-egalitarian) view of society.

Given the critical role of the similarity and overlapping between the Self and outgroup, it is not surprising that we also disclosed a general impact of the changes of interpersonal closeness on the changes of implicit racial associations prior to and following a social interaction. This finding seems to suggest that the reduction of perceived distance between the participants and the confederate experimenter, in terms of increased dIOS, was overall associated with the reduction of implicit racial bias, in terms of decreased dIAT: the closer the distance, the lower the racial bias. However, the significant interaction between closeness and implicit bias indicated that the linear association between dIOS and dIAT was statistically significant in the context of a neutral, but not a cooperative, presence. This suggests that perceived closeness has a mediating role in reducing the initial racial bias when the interaction is not cooperative, namely the effect of embodying a Black avatar in terms of decreased IAT depends on whether participants perceived the other more close to themselves or not. More interestingly, controlling for the different impact of perceived psychological closeness on the two groups, we found decreased IAT scores only in the cooperative, but not in the neutral group, with lower implicit racial scores after the cooperative interaction. It is interesting to note here that Hasler et al. (2017) in the case of a neutral interaction (simple embodiment) reported no change in implicit racial bias. Hasler et al. (2017) study is indeed different from previous work on RHI and virtual embodiment since there was an actual social exchange that participants could have experienced as negative or positive, depending on their individual preferences. The authors showed that, for those embodied in a Black avatar, the extent to which they liked their virtual confederate was associated with a reduction in implicit bias. The results of the current study line up with Hasler et al. (2017) findings: in the neutral group we did not find evidence of reduction in the IAT scores, nonetheless, the changes in initial associative bias were associated with the changes in perceived closeness. Although perceived closeness, as measured by the IOS scale, could be considered as an indirect index of likeability, the two dimensions are correlated but independent (Aron et al., 1992; Launay and Dunbar, 2015; Ventre-Dominey et al., 2019). Here we point out that in our study we considered the changes in perceived distance (i.e., delta) as a variable of interest. Taking into account only either the initial impression of the other person (before interaction) or the subsequent assessment (after interaction without controlling for baseline) would not have provided a fully reliable measure of a complex social dimension such as perceived interpersonal closeness. Hence, measuring the pre-post changes in psychological closeness could be a more fruitful strategy to control for and test the influence of such a variable on implicit racism. In this regard, we should also emphasize the absence of the association between changes in interpersonal closeness and the racial bias within the context of a cooperative interaction. This indicates that the reduction in the initial racial scores cannot be merely explained, for instance, by higher psychological closeness (or higher likability) after cooperating with the other person. Put differently, the effect of cooperation in reducing racial implicit associations is independent of psychological closeness. One could thus speculate that having people to experience a cooperative VR interaction could have a positive effect in reducing implicit racial bias that can potentially brush off detrimental effects derived by, for example, the first impression of the other person and other forms of in-group/out-group prejudice. These findings are in keeping and provide further support to studies within the two major lines of research that exploit cooperation to reduce prejudice: the contact hypothesis (Allport, 1954; for meta-analysis see Pettigrew and Tropp, 2006, 2008) and the recategorization approaches (Bettencourt et al., 1992; Brewer, 2000; Gaertner and Dovidio, 2014) inspired by social categorization and social identity theories (Tajfel, 1970; Miller and Brewer, 1986). Obviously, the beneficial effects of cooperation in reducing prejudice is not a novelty in social science (see, for instance, the pioneering Sherif et al.’s (1961) Robbers Cave experiments or Eliot Aronson’s “Jigsaw classroom” technique by Aronson et al. (1978); see Paluck and Green (2009) for a review). However, here we demonstrate that these beneficial effects can be achieved through VR, a technique offering countless and perhaps not totally explored advantages in this field. Capitalizing on social psychology findings, we therefore encourage scholars from different domains to further explore whether and under which conditions a given type of social manipulation for reducing racial bias could be efficiently transposed in VR.

Here we turn to discussing the hypothesis that the traits of empathy and political attitudes could contribute to implicit attitudes about race. In addition, we discuss the relationship between some political variables and dispositional empathy, as this may help developing models that take these factors into account as mediators, or moderators of implicit racial bias. We first examined whether political attitudes and the single scales of IRI could explain implicit racial bias in our entire sample (before any experimental manipulation). The results suggest that dispositional empathy, indeed, plays a relevant role in predicting implicit racial bias, as measured by the IAT, being empathic concern, perspective-taking, and personal distress all significant predictors of the observed implicit racial scores. Specifically, people with the tendency to respond with sympathy and compassion, as well as those who tend to adopt the psychological points of view of others, showed smaller implicit racial bias (*negative* association with IAT). Conversely, the association between personal distress and the IAT scores was *positive*: people with stronger feelings of personal unease and discomfort in reaction to the emotions of others showed also a stronger implicit bias. Although psychological research has confirmed that empathy is a prejudice-reducing factor (Batson et al., 1997; Galinsky and Moskowitz, 2000; Todd et al., 2011; Pashak et al., 2018), social neuroscience has focused on how empathy constrains motor and pain resonance, showing reduced motor activation when observing racial outgroup members’ actions (Gutsell and Inzlicht, 2010) or racial outgroup members’ pain (Xu et al., 2009; Avenanti et al., 2010). This reduced response might be a manifestation of a more fundamental and general bias in response to outgroups. In this regard, a study suggested that prejudice toward different target groups may be represented as a general prejudice factor (Bäckström and Björklund, 2007). More specifically, the authors showed that a large share of the individual differences, in terms of variance, in the level of general prejudice was accounted by empathy (as assessed by the IRI scale, Davis, 1983), social dominance orientation (Pratto et al., 1994), and right-wing authoritarianism (Altemeyer, 1988). However, this study assessed general prejudice by e*xplicit* questionnaires about classical and modern racism (McConahay, 1986; Akrami et al., 2000) and considered empathy as unidimensional, neglecting its multidimensional assessment in four scales. Our findings importantly add to this field, as we demonstrate an association between *implicit* racial bias and dispositional empathy. Here we suggest that the traits of empathy could have a twofold effect on racial attitudes. On one hand, as empathic concern and perspective-taking are negatively associated with lower implicit racial bias, the two aspects could be a protective factor against prejudice (see also McFarland, 2010). On the other hand, higher levels of personal distress could be a risk factor. In fact, neuroscientific research has shown that, when Caucasian people are shown Black faces there is an initial activity in the amygdala, followed by the dorsolateral prefrontal cortex (dlPFC) and the anterior cingulate cortex (ACC), brain areas that modulate and regulate amygdala activity (Cunningham et al., 2004). Richeson et al. (2003), see also Kubota et al., 2012, for a review) demonstrated that people with the highest IAT scores also had the highest activation levels in the dlPFC and ACC regions after exposure to Black faces. Hence, elevated levels of personal distress (as measured by the IRI scale) could be associated with the initial activity of the amygdala, a response they try suppressing to avoid showing any explicit racial behavior.

In our view, empathy, social dominance, and implicit racial bias are intimately intertwined. This intimacy could explain the third interesting novel finding of this study, the correlation between political attitudes and empathy: The more participants reported to be left-wing voters, the higher their perspective-taking and empathic concern scores. To the best of our knowledge, this is the first account of such a relationship. Only a few studies have described how empathy could be a mediator of the relation between social dominance orientation, authoritarianism, and expressions of racism and sexism (Bäckström and Björklund, 2007; Nicol and Rounding, 2013). To note, these studies did not explicitly ask political attitudes, as they measured right-wing authoritarianism, which is more related to uncritical subjection to authority, conventionalism, and traditionalism (Duckitt and Bizumic, 2013). Instead, here we captured political attitudes by means of a single-item self-report, an explicit approach that might be useful in future work. When considering the neural correlates of political orientation, a pioneering fMRI study disclosed that greater liberalism was associated with increased gray matter volume in the ACC, whereas greater conservatism was associated with increased volume of the right amygdala (Kanai et al., 2011). In fact, ACC not only is involved in amygdala regulation, but it is also a crucial area for empathizing, being linked to affective empathy and perspective-taking (Banissy et al., 2012). Some complementary evidence also suggests that individual differences in social dominance orientation predict neural response in the insula and ACC (Chiao et al., 2009). The idea of an insula-amygdala-ACC interplay as a shared neuro-cognitive substrate of empathy *and, therefore*, political attitudes and social orientation, is fascinating, yet, it remains mere speculation and further studies need to address this complex scenario that we are just starting to figure out.

Before concluding, we consider some potential limitations of this study. To separately assess the effect of embodying different races, one would have included White participants embodying a White. We did not include this situation because our aim was to assess the role played by cooperation on racial biases, and it does so by comparing a neutral condition, in which the confederate’s Black avatar was present but inactive, to a cooperative condition, in which the same confederate’s Black avatar was present and helpful. In addition, when such a control for race was present, it did not reveal any significant reduction in the IAT scores when participants embodied a White avatar (e.g., Peck et al., 2013; Banakou et al., 2016). Future studies will specify to what extent cooperating in a Black body has an additive effect in reducing racial bias, as compared to cooperating in an avatar of the same ethnicity. Related to this, another interesting contrast would include two “non-VR embodiment” conditions, in which participants would (or not) cooperate without being in a Black body. These additional conditions would allow us to assess and quantify to what extent cooperating in a Black body has an enhancing effect in reducing racial bias. Second, participants saw the confederate experimenter in VR as Black, but they knew she was in reality Caucasian. Knowing that the other person is not actually Black could have reduced the strength of our manipulation. Third, our regression model and correlation analyses were carried out in a relatively small sample (*N* = 40). Other scholars will hopefully apply similar analyses to bigger samples. Therefore, this study should be considered as having generated new experimental hypotheses for future work rather than as a conclusive result.

## Conclusion

Our findings critically contribute to the study of prejudice within the neuroscientific and social cognition field and have practical implications. First, we revealed the effects of a cooperative interaction within a VR scenario where embodying a Black virtual body resulted in a reduction of the implicit racial bias against Black people. We also showed that, when the interaction is not cooperative the effects following the embodiment of a Black avatar depend on the perceived distance with the other person: only when people perceive the other person as closer to themselves does their initial racial bias diminish. Furthermore, this study emphasizes the key role of an important variable in protecting from prejudice: empathy–the higher the emphatic concern and perspective taking skill, the lower the initial bias. Not last, the study explores the relationship between political attitudes and empathy, encouraging further work to assess how and to what extent these and other socio-cognitive and political factors contribute to or reduce racial attitudes and discriminatory behavior. These findings are crucial not only for the theoretical understanding of these phenomena but also for the applicative use of the VR technique. Indeed, prejudice continues to have significant and pervasive consequences on virtually any area of our society: physical and mental health, employment, education, healthcare, housing, and financial systems (e.g., Pager and Shepherd, 2008; Pascoe and Smart Richman, 2009). Effective approaches are needed to reduce prejudice (explicitly and implicitly assessed) and one challenge is the limited number of people having access to specific *ad hoc* interventions. For breaking down barriers, a promising direction for intervention development is putting one into a situation of “being” a member of the out-group through virtual embodiment. While further studies with empirical assessment of prejudice reduction strategies and controls over socio-cognitive variables affecting interventions are needed, we suggest that cooperative interactions in VR may add to the beneficial effects of intergroup contact.

## Data Availability Statement

All datasets generated for this study are included in the article/Supplementary Material.

## Ethics Statement

The studies involving human participants were reviewed and approved by Inserm Ethics Board (IRB00003888). The patients/participants provided their written informed consent to participate in this study.

## Author Contributions

AF, IP, and MS provided the concept. AL, GV, CD, EK, and RS provided software, hardware, and electronic solutions for the VR scenario and the Neuro-Immersion Lab. DB provided the scripts for running IAT. AL and IP collected and processed the data, and analyzed by IP. IP, AL, and DB drafted the manuscript. IP, DB, AF, and MS revised the manuscript. All authors approved the manuscript.

## Conflict of Interest

The authors declare that the research was conducted in the absence of any commercial or financial relationships that could be construed as a potential conflict of interest.

## Footnotes

1 www.oculus.com/support

2 https://www.noitom.com/

3 https://neuronmocap.com

## Supplementary Material

VIDEO S1 Illustrates the familiarization phase.

VIDEO S2 Illustrates the Neutral condition.

VIDEO S3 Illustrates the Coop condition of the experiment.

ANALYSES S1 Separate analysis of the Iat and Ios scales.
