# Supplementary material for: Exploring the Effect of Cooperation in Reducing Implicit Racial Bias and Its Relationship With Dispositional Empathy and Political Attitudes
Source: Front Psychol. 2020 Oct 28;11:510787. doi: 10.3389/fpsyg.2020.510787 (PMC7655932; doi:10.3389/fpsyg.2020.510787)
Supplement: ANALYSES S1 — Separate analysis of the Iat and Ios scales. [file Data_Sheet_1.docx]

**Exploring the effect of cooperation in reducing implicit racial bias and its relationship with dispositional empathy and political attitudes**

**Ivan Patané *^#1,2,3^ , Anne Lelgouarch^#1,2,3^, Domna Banakou^#4,5^ ,  Gregoire Verdelet^1,2,3^Clement Desoche^1,2,3^, Eric Koun^1,2,3^, Romeo Salemme^1,2,3^, Mel Slater^4,5^ , Alessandro Farnè^1,2,3 ,6^**

*Supplementary analyses*

*IAT*

To test for reduction of the IAT scores in the two groups, we first calculated the change in IAT prior to and following the VR exposure (dIAT = postIAT - preIAT) and then contrasted dIAT against 0. The one-sample two-tailed t-test against 0 was not significant (t(19) = 0.21, p = 0.84) in the Neutral group, while there was a tendency toward a significant reduction in the Coop group (t(19) = -1.79, p = 0.089).

*IOS*

To test whether our experimental manipulation differently affected psychological closeness toward the confederate experimenter between the two groups, we computed the changes in IOS before and after the VR exposure (dIOS =  postIOS - preIOS) and submitted  dIOS scores to one-sample two-tailed t-tests against 0. Analysis showed a significant dIOS increase in both groups (Neutral t(19) = 2.65, p = 0.016; Coop t(19) = 6.24, p <.001).
